# How Do Environmental Regulation and Environmental Decentralization Affect Regional Green Innovation? Empirical Research from China

**DOI:** 10.3390/ijerph19127074

**Published:** 2022-06-09

**Authors:** Jing Tang, Shilong Li

**Affiliations:** 1 School of Management Science and Real Estate, Chongqing University, Chongqing 400044, China; tangjingcqu@126.com; 2 Center for Construction Economy and Management, Chongqing University, Chongqing 400044, China

**Keywords:** environmental regulation, environmental decentralization, regional green innovation, fixed-effects model, threshold panel model

## Abstract

Green innovation is vital in transforming China’s economic development from high speed to high quality. Environmental regulation plays an important role in stimulating regional green innovation, and appropriate environmental decentralization is the institutional basis to consolidate the innovation compensation of environmental regulation. Clarifying the relationship among environmental regulation, environmental decentralization, and green innovation is of great theoretical and practical significance for regional environmental management and green innovation development. This paper incorporates environmental regulation, environmental decentralization, and regional green innovation into the same analytical framework and constructs a fixed-effects model and a threshold panel model to empirically examine the intrinsic relationship between them based on panel data of 30 Chinese provinces from 2006 to 2015. The estimation results indicate that environmental regulation has a positive impact on regional green innovation, which is greater in developed regions than in underdeveloped regions. Environmental decentralization plays a negative role in regional green innovation, with underdeveloped regions being affected to a greater extent. The impact of environmental regulation on regional green innovation shows a threshold characteristic with the change of the degree of environmental decentralization, while the green innovation utility of environmental regulation gradually decreases with the increase of the degree of environmental decentralization.

## 1. Introduction

China’s economic development has been soaring since its reform and opening up, creating a world-renowned growth miracle. However, the long-standing extensive and high-speed economic development model has exacerbated energy consumption and environmental pollution and become a bottleneck in limiting the green and coordinated development of the economy and society [1,2]. According to the British Petroleum World Energy Statistical Yearbook (2021), global energy consumption fell by 4.5% in 2020 compared to the previous year, creating the largest decline since the end of World War II. However, China’s consumption of fossil fuels and renewable energy had inverse growth, with natural gas use increasing by almost 7% and oil demand increasing by 220,000 bpd, making it one of the few major economies to see an increase in energy demand. Facing the growing exacerbation of both energy demand and ecological damage, it has become increasingly vocal about accelerating the transformation of the economy from high speed and extensive development to high quality and green development [3,4,5]. Served as an important driving force to improve energy utilization and mitigate environmental pollution, the green innovation has become an inevitable choice for “win-win” economic and environmental development [6,7].

The negative externality of environmental pollution and the positive externality of green innovation lead to an increase in the inertia of local enterprises to undertake environmental management and green innovation [8]. On the one hand, local enterprises pay no cost for the extraction of natural resources; on the other hand, the benefits of environmental management and green innovation undertaken by local enterprises are shared by all economic parties, while the costs are borne alone. Therefore, internalizing the externality problem is the key to incentivizing local enterprises to undertake pollution prevention and green innovation [9,10]. Serving as the primary instrument to internalize pollution externalities, the mechanism of influence of environmental regulation on regional green innovation is receiving widespread attention from scholars [11,12,13,14]. The Chinese government has also issued a series of environmental regulatory policies in recent years, such as the Environmental Protection Law of the People’s Republic of China, the Environmental Protection Tax Law of the People’s Republic of China, and the Regulations on the Administration of Emission Permits, with a view to promoting regional environmental governance and green and innovative development [15]. The core of the environmental management system lies in the allocation of authority over environmental management affairs among different levels of government, that is, the problem of environmental decentralization. This context indicates that environmental decentralization may be a key factor affecting the effect of environmental regulation on green innovation [16,17,18]. So, what is the relationship between environmental decentralization, environmental regulation, and regional green innovation? Does the environmental decentralization really weaken the green innovation effect of environmental regulation? If so, under what conditions does environmental decentralization have a dampening effect? Clarifying the above questions will not only help to optimize the environmental decentralization system, but also has important practical implications for the realization of regional environmental governance and high-quality green development.

Based on the institutional environment during China’s market economy transition period, this study explores the mechanisms of environmental regulation and environmental decentralization on regional green innovation at the regional level. There are three main contributions of this study to the literature. First, environmental regulation, environmental decentralization and regional green innovation are analyzed under the same framework. Thus, the research conclusions contribute to a more comprehensive understanding of the internal mechanisms by which environmental regulation plays a role in green innovation. Second, this study extends the theoretical extension of Porter’s hypothesis and uses a fixed-effects model to analyze the impact of various types of environmental decentralization on green innovation in different regions, providing a theoretical basis for different regions to develop differentiated environmental decentralization strategies. Finally, the moderating effect of environmental decentralization on the green innovation effect of environmental regulation is explored, and the effective range of its role is determined and tested based on the threshold model, thus contributing to the high-quality and green development of the regional economy.

The rest of the paper is organized as follows. Section 2 provides a brief literature review. Section 3 performs a mechanism analysis of environmental regulation, environmental decentralization, and regional green innovation. Section 4 explains the estimation model and data used in this study. Section 5 presents empirical results and discussion. Section 6 provides conclusions and related policy implications.

## 2. Literature Review

Current and expected government regulation is particularly important with regard to pushing firms to engage in green innovation [19,20], and its green innovation effect has led to a debate between the “facilitation” and “disincentive” arguments, i.e., whether the “innovation compensation” effect of environmental regulation prevails. The “promotion theory” studies the effect of environmental regulation on green innovation from a dynamic perspective, and the most central explanatory theory is the “Porter hypothesis” [21]. It suggests that under free trade and economic globalization, companies in developed countries, which have stricter environmental regulations than developing countries, are at a disadvantage in price competition. Here, firms that successfully innovate to reduce polluting emissions can reduce the burden of regulatory costs and thus compete on price favorably with other firms [22,23]. The promotion effect of environmental regulation on green innovation has been empirically tested by many scholars based on this hypothesis [24,25,26,27]. The “disincentive theory”, based on neoclassical economics, states that increasing the environmental regulation intensity will lead to an increase in the pollution control cost for producers, resulting in their reduction on green innovation inputs, thus inhibiting the development of regional green innovation [28,29]. In addition, some scholars have also suggested that the green innovation effect of environmental regulation has non-linear characteristics [30,31,32], which are influenced by factors such as government subsidies [33], R&D investment [34], industrial structure [35], and foreign investment [36].

Although the conclusions of the above studies are mixed, they are mostly based on the theoretical perspective of welfare economics, which assumes that local governments always seek to maximize social welfare. However, environmental federalism and public choice theory reject the assumption of “welfare government” and propose the assumption of “economic man” in the political market, arguing that local governments have the characteristic of maximizing their own interests [37]. The incentive incompatibility between local governments’ behavioral preferences and environmental governance goals often leads to selective enforcement of regulations [38], and environmental decentralization reforms are the main cause of local government behavioral alienation. Accordingly, plenty of studies have explored the impact mechanisms of environmental decentralization on regional green innovation. Some scholars have argued that environmental decentralization is conducive for local governments to strengthen environmental regulation and force enterprises to engage in green technological innovation, thus promoting the development of regional green innovation [39,40,41]. While others have suggested that excessive environmental decentralization can lead to strategic interactions and a “race to the bottom” in the environmental regulation behavior of local governments, weakening the incentive to internalize pollution and thus inhibiting regional green innovation [42].

There are diversity characteristics in environmental affairs management, and scholars have classified environmental decentralization into environmental administration decentralization, environmental supervision decentralization, and environmental monitoring decentralization according to their functions [43]. Different types of environmental decentralization have differential effects on green innovation in different regions [44,45,46]. Mohamed et al. used panel quantile regression to confirm the U-shaped effect of environmental decentralization on regional green growth [47]. Wu, H. et al. suggested that environmental decentralization and environmental administrative decentralization have a facilitating effect on regional green development, while environmental supervision and monitoring decentralization have a negative effect [48]. Zhang, W. et al. found that environmental decentralization promotes green technology innovation after inhibition, and in the long run, environmental decentralization in developed regions and low-emission regions is more conducive to green technology innovation [49].

Despite the increasing literature on environmental regulation, environmental decentralization, and green innovation, research in this area still has some limitations. First, the previous literature did not incorporate environmental regulation, environmental decentralization, and green innovation into the same analytical framework for theoretical and empirical research. Second, previous studies ignored the differential impact of different environmental decentralization on green innovation in different regions, which is not conducive to the development of differentiated environmental decentralization strategies. Finally, previous literature neglected the moderating effect of environmental decentralization on the green innovation effect of environmental regulation.

## 3. Mechanism Analysis and Hypothesis

### 3.1. Environmental Regulation and Green Innovation

As an essential tool for governments to combat environmental pollution, the impact of environmental regulation on green innovation can be divided into two aspects: macro regulation and micro autonomy. At the macro level, strict environmental regulation policies help to raise people’s awareness of green consumption as well as increase enterprises’ demand for green production, thus promoting the development of regional clean industries and realizing a regional green industrial structure. In addition, environmental regulation raises the barriers to entry for foreign investment and enhances the quality of foreign investment through market access, pollution taxes, and green product certification [50], which in turn brings advanced production management technologies to the investing region and promotes local green innovation development. At the micro level, firms are forced to pay more attention to the development of green innovation to reduce the long-term costs of pollutant emissions and improve core competitiveness, thus generating an “innovation compensation effect” and driving the development of regional green innovation. From this Hypothesis 1 is proposed:

**Hypothesis** **1.** *Environmental regulation may promote regional green innovation.*

### 3.2. Environmental Decentralization and Green Innovation

Fiscal decentralization theory states that the decentralization of environmental management affairs facilitates local governments to develop and implement locally adapted environmental policies, thereby improving the performance of regional environmental governance and innovation [51]. However, China’s unique government management model and GDP-focused promotion assessment mechanism have reshaped the incentives of local government officials. As the marginal value generated by productive investments outweighs the social benefits of environmental expenditures, local governments are driven by promotion incentives to pursue rapid economic growth at the cost of environmental pollution and excessive energy consumption. This unsustainable growth model leads to serious negative externalities of environmental pollution, further hindering the development of regional green innovation.

Market fragmentation due to environmental decentralization may trigger distortions in resource allocation and homogeneous industrial structure, which makes economies of scale difficult to achieve and leads to energy efficiency losses [52]. In addition, local protectionism induced by environmental decentralization inhibits the development of regional green innovation. First, local protectionism discourages the free flow of production factors and the introduction of technology-intensive foreign investment, which in turn hinders the rational allocation of resources and the diffusion of technology [53]. Second, the existence of local protectionism may affect the functioning of market mechanisms and lead to the slow development of high-productivity, low-energy consuming industries. Thus, Hypotheses 2a and 2b are proposed:

**Hypothesis** **2a.** *Environmental decentralization may hinder regional green innovation.*

**Hypothesis** **2b.** *The inhibitory effect of Environmental decentralization in underdeveloped regions may be larger due to the local protectionism.*

### 3.3. Environmental Regulation, Environmental Decentralization and Green Innovation

**Figure 1:**
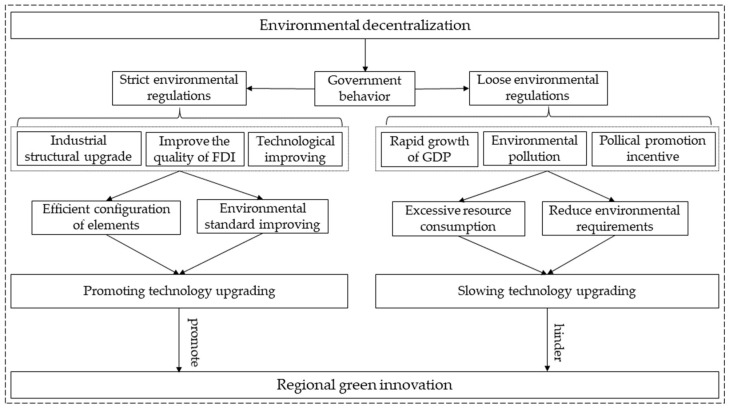
The mechanism analysis of environmental regulation, environmental decentralization, and green innovation.

Environmental decentralization reflects the delegated relationship of environmental affairs between central and local governments. Local governments are the main agent carrying out environmental regulation functions, and their behavior under the environmental decentralization system is the decisive factor influencing the effectiveness of environmental regulation, and its specific mechanism of action is shown in Figure 1. Remarkable differences can be found among the impacts of different types of environmental decentralization on environmental regulation effect. Environmental monitoring decentralization can give full play to the information advantages of local governments, enabling them to formulate and implement appropriate environmental regulation policies, which helps to achieve the effective allocation of regional resource factors by promoting industrial structure upgrading and improving foreign investment quality, thus ultimately promoting green innovation development. Environmental administration and supervision decentralization gives local governments more power to determine the level of environmental regulation. In pursuit of sustained and stable economic growth, local government officials may gradually relax the level of environmental regulations on highly polluting and profitable tax enterprises [54]. Under this context, energy waste and environmental pollution have become the norm for companies to reduce operating costs and increase production revenue. The incentive for enterprises to engage in green innovation will gradually weaken, leading to the development of regional green innovation being hindered. Hypotheses 3a and 3b are proposed:

**Hypothesis** **3a.** *Environmental administration and supervision decentralization may weaken the green effect of environmental regulation.*

**Hypothesis** **3b.** *Environmental monitoring decentralization may strengthen the green effect of environmental regulation.*

## 4. Research Design

### 4.1. Model Construction

#### 4.1.1. Benchmark Model Construction

Fixed-effects and random-effects models are suitable for analyzing panel data [55,56]. In order to investigate the impact mechanism of environmental regulation and environmental decentralization on regional green innovation, this study selects fixed-effects model based on the Hausman test results. Model (1) was constructed to test the relationship between control variables and regional green innovation, and model (2) was constructed to test the relationship between environmental regulation and regional green innovation. Model (3) was constructed to test the relationship between environmental decentralization and regional green innovation.

(1) $$\begin{array}{c} GI_{i,t}=\alpha_{0}+\alpha_{1}Control_{i,t}+\mu_{i}+\delta_{t}+\varepsilon_{i,t} \end{array}$$

(2) $$\begin{array}{c} GI_{i,t}=\alpha_{0}+\alpha_{1}ER_{i,t}+\alpha_{2}Control_{i,t}+\mu_{i}+\delta_{t}+\varepsilon_{i,t} \end{array}$$

(3) $$\begin{array}{c} GI_{i,t}=\alpha_{0}+\alpha_{1}ED_{i,t}+\alpha_{2}Control_{i,t}+\mu_{i}+\delta_{t}+\varepsilon_{i,t} \end{array}$$

where $GI_{i,t}$ is the logarithm of the number of regional green patent applications, $ER_{i,t}$ is the intensity of environmental regulation, $ED_{i,t}$ is the intensity of environmental decentralization, $Control_{i,t}$ are a set of control variables, $\alpha_{0}$ is the constant term, $\alpha_{1}$ and $\alpha_{2}$ are the correlation coefficients of the explanatory and control variables respectively, $\mu_{i}$ and $\delta_{t}$ are the dummy variables for individual fixed effects and time fixed effects respectively, and $\varepsilon_{i,t}$ is a random disturbance term.

#### 4.1.2. Moderating Effect Model Construction

Environmental decentralization is introduced as a moderating variable in the baseline model to test whether the relationship between environmental regulation and regional green innovation differs according to the intensity of environmental decentralization. To this end, model (4) was constructed by adding environmental decentralization ($ED_{i,t}$) and the cross product of environmental regulation and environmental decentralization ($ED_{i,t}\times ER_{i,t}$) to model (2) to test the moderating role of environmental decentralization in environmental regulation and regional green innovation.

(4) $$\begin{array}{c} GI_{i,t}=\alpha_{0}+\alpha_{1}ER_{i,t}+\alpha_{2}ED_{i,t}+\alpha_{3}ED_{i,t}\times ER_{i,t}+\alpha_{4}Control_{i,t}+\mu_{i}+\delta_{t}+\varepsilon_{i,t} \end{array}$$

On the basis of Equation (2), a double-threshold regression model with regional green innovation as the explained variable, environmental regulation as the core explanatory variable, and environmental decentralization as the threshold variable is developed in this paper, to explore the non-linear effects of environmental regulation on regional green innovation when environmental decentralization is at different thresholds. The model is set out as follows:

(5) $$\begin{array}{c} GI_{i,t}=\alpha_{0}+\alpha_{1}ER_{i,t}I\left(ED_{i,t}\leq \gamma_{1}\right)+\\ \alpha_{2}ER_{i,t}I\left(\gamma_{1}\leq ED_{i,t}\leq \gamma_{2}\right)+\alpha_{3}ER_{i,t}I\left(ED_{i,t}\geq \gamma_{2}\right)+\beta_{m}Control_{i,t}+\varepsilon_{i,t} \end{array}$$

where *i* and *t* denote province and time, respectively; *I*(.) is the indicator function; $ED_{i,t}$ is the threshold variable; $\gamma_{1}$, $\gamma_{2}$ are the thresholds to be estimated; $\alpha_{1}$, $\alpha_{2}$, $\alpha_{3}$ are the impact coefficients of the explanatory variables in different intervals; $\beta_{m}$ is the parameter to be estimated for each control variable, and $\varepsilon_{i,t}$ is the random disturbance term.

### 4.2. Variable Selection

#### 4.2.1. Green Innovation (GI)

There are three main types of methods for measuring green innovation: first, adopting data envelopment analysis (DEA) and the principal component method to measure green innovation efficiency [57,58], second, using green patent statistics to measure [59,60,61], and third, measuring from both process and product levels [62,63]. Since green patent statistics mainly reflect the innovation in resource conservation, energy efficiency, pollution prevention and control, and cleaner production, they can more intuitively measure the overall level and scale of green innovation activities in a region compared to other analysis methods [64]. This study takes reference from Wurlod et al. and adopts the logarithm of green patent applications to characterize green innovation.

#### 4.2.2. Environmental Decentralization (ED)

Environmental decentralization is a mechanism for the division of environmental management and affairs based on the separation of powers. Qi et al. [65] have pointed out that institutions and staffing are the carriers through which governments realize their public services and functions, and the staffing of environmental agencies at different levels of government reflects, to a certain extent, the distribution of power over environmental affairs at different levels of government. Moreover, the changes of the staff proportion in environmental agencies at different levels of government better reflect changes in the environmental management system. Therefore, this study draws on the indicator selection method of Ran et al. [66] and utilizes the staff distribution characteristics of environmental protection departments at different levels of government to portray environmental decentralization intensity. The specific environmental decentralization measurement formula is as follows:

(6) $$\begin{array}{c} ED_{i,t}=\left[\frac{Lep_{i,t}/Pop_{i,t}}{Nep_{t}/Pop_{t}}]\times [1-\left(GDP_{i,t}/GDP_{t}\right)\right] \end{array}$$

where $Lep_{i,t}$, $Pop_{i,t}$ and $GDP_{i,t}$ denote the number of personnel in the environmental protection system, the size of the regional population, and the gross domestic product in province *i* and in year *t*, respectively. and $Nep_{t}$, $Pop_{t}$, and $GDP_{t}$ denote the number of personnel in the national environmental protection system, the size of the national population, and the national gross domestic product in year *t*, respectively. The environmental decentralization indicators are effectively deflated by adding the economic scale reduction factor [$1-\left(GDP_{i,t}/GDP_{t}\right)$]. In addition, this study further subdivides environmental decentralization into environmental administrative decentralization, environmental supervision decentralization, and environmental monitoring decentralization, and the three subdivided indicators are measured similar to Equation (5) by simply replacing the number of personnel in the environmental protection system with the corresponding number of personnel in the subdivided indicators.

#### 4.2.3. Environmental Regulation (ER)

At present, there are several approaches to measuring environmental regulation in academic circles: The first is the single indicator approach, which classifies environmental regulation into command-and-control, market-incentive, and public-participation types according to different participants and measures them by indicators such as the number of environmental administrative penalties, the amount of pollution control, and the number of environmental petitions, respectively [67,68]. The second is the composite indicator method, which uses the entropy method or linear weighting method to construct environmental regulation intensity indicators to reflect the regional environmental regulation level [69,70]. However, single indicators do not provide a comprehensive measure of the overall intensity of environmental regulation and there is a large margin of error in the statistical caliber of the composite indicator or in the process of normalization and calculation. Therefore, this study draws on the approach of Kheder, S. B. et al. [71] to measure the intensity of regional environmental regulation by using *GDP*/*energy*, where *GDP* represents regional gross domestic product and is deflated using 2006 as the base period, *energy* represents total regional energy consumption. This indicator measures the true effect of a range of environmental laws and regulations.

#### 4.2.4. Control Variables

Regions with high level of economic development tend to have strong innovation capacity. Urbanization and industrial structure upgrading accelerate the concentration of capital and technology in the region, and foreign investment provides a source of funding for regional green innovation. In this study, with reference to relevant literature, the following indicators are selected to control for the above variables: Regional economic development (RGDP), expressed as the logarithm of GNP per capita by province and deflated using 2006 as the base period; the regional urbanization rate (UR), measured by the proportion of urban population in each province; the industrial structure (IND), expressed as the logarithm of the value added of tertiary industries in each province; and the total foreign investment (TFI), expressed as the logarithm of the total investment by foreign enterprises in each province, and deflated using 2006 as the base period.

### 4.3. Variables Description and Data Source

The descriptive statistics for the relevant variables in this study are shown in Table 1. The mean value of GI is 2785 and the standard deviation is 4180, which indicates that the level of green innovation varies greatly from region to region. The minimum value of GI is 14 and the maximum value is 28,049, indicating that there is regional heterogeneity in the level of green innovation, with developed regions likely to develop faster than underdeveloped regions. RGDP has the largest standard deviation among other variables, which is due to the great differences in the level of economic development of the provinces. The range of values for all variables is within the normal range, which confirms the reliability of the data.

In this study, 30 provincial administrative units (excluding Tibet and Hong Kong, Macao and Taiwan) in China from 2006–2015 were selected as the sample for examination by considering the following reasons. First, in 2006, the State Environmental Protection Administration (SEPA) established five direct inspection centers and eleven direct enforcement and supervision agencies to inspect local governments’ interference with environmental enforcement, thereby increasing the central government’s authority in environmental regulation. This has contributed to a change in the distribution of regulatory authority between central government and local governments, and to a certain extent has increased central government’s regulatory powers, especially its power to monitor and enforce the law. Second, it has been only about five years since the launch of vertical management reform of sub-provincial environmental protection agencies for monitoring and supervising in 2016. The empirical material does not yet support testing the impact of this reform on the distribution of the regulatory authority between central government and the local authorities and the impact on green innovation. Thus, selecting the data from 2006 to 2015 can better reflect the impact of China’s environmental decentralization system on green innovation, which is not only a summary of previous policies, but also provides a theoretical basis for future policy formulation. The data related to environmental decentralization come from the China Environment Yearbook, the data related to R&D investment and personnel come from the China Science and Technology Statistical Yearbook, the data related to green patents come from the CNRDS database, and the data related to other variables involved in this paper come from the China Environment Statistical Yearbook, the China Statistical Yearbook, and the China Energy Statistical Yearbook. For the problem occurring where some of the data were missing, they were smoothed out according to the trend presented by the data in this study.

## 5. Empirical Results and Discussion

### 5.1. Overall Estimated Results

This study uses Stata 16.0 to estimate Models (1), (2) and (3) by using robust standard errors, and the regression results are shown in Table 2. It can be seen that the estimated coefficient of environmental regulation is positive and passes the 1% significance level, indicating that environmental regulation has a significant positive impact on regional green innovation, and the green innovation promotion effect of environmental regulation is prominent. This is consistent with Hamamoto‘s findings [72] and validates Hypothesis 1. The estimated coefficients for environmental decentralization, environmental administrative decentralization, and environmental supervision decentralization are all significantly negative and pass the robustness test, confirming the truth of Hypothesis 2a. Comparing the coefficients of each type of environmental decentralization, it is observed that the negative effect of environmental administrative decentralization on regional green innovation is greater, with an impact coefficient of −0.147. The reason for this is that environmental administration mainly involves the formulation of relevant environmental protection plans and the approval of administrative permits. It has given local governments more power to determine the level of regional green development. Local governments may relax their requirements for green innovation to pursue high economic growth [73], thus hindering the development of regional green innovation.

By observing the regression results of other control variables, it can be seen that the estimated coefficients of the economic development (RGDP), the urbanization rate (UR), and the advanced industrial structure (IND) are all significantly positive. The increase in the level of economic development provides good material foundation for regional green innovation; rapid urbanization creates a gathering effect of labor and technical personnel [74], which provides manpower for regional green innovation; the rapid upgrading of industrial structure puts forward higher requirements for green development for local enterprises [75], thus accelerating the green innovation by local enterprises. The estimated coefficient of total foreign investment (TFI) is significantly negative, indicating that foreign investment has a suppressive effect on regional green innovation, confirming the findings of HARRISON A et al. [76]. Although foreign investment has shown a gradual increase every year, most foreign investors tend to transfer low-tech industries with high pollution and energy consumption to the host country, further deteriorating the host country’s environment and creating a “pollution paradise” effect, which is not conducive to enhancing the development of green innovation in the host country [77].

### 5.2. Regional Estimated Results

Due to the large differences in economic strength and policy conditions across regions in China, in order to verify whether there is regional heterogeneity in the empirical results, most previous studies have divided the classical economic regions based on the geographical location of each province, which in turn splits China into three major regions: the east, the center, and the west [78]. However, such division does not effectively reflect the economic differences among provinces. This study draws on the approach of Zhang, W. et al. [49], and uses the GDP per capita of each province in 2020 as a classification criterion, those greater than the national average are classified as developed regions, while those less than the national average are classified as underdeveloped regions. It then discusses in depth the impact of environmental decentralization on the regional heterogeneity of green innovation.

The empirical results in Table 3 show that the estimated coefficient of environmental regulation is significantly positive in both developed and underdeveloped regions. Notably, the estimated coefficient is much larger in developed regions than in underdeveloped regions, indicating that the “push-back” effect of environmental regulation on green innovation is more pronounced in developed regions. It follows that compared to developed regions, underdeveloped regions have limited economic power, and when the intensity of environmental regulation increases, local governments are unable to balance the effective synergy between economic development and green innovation, thus reducing their investment in green patent research and development, resulting in slower development of green innovation. The estimated coefficient of environmental decentralization is significantly negative across regions, with a larger level in underdeveloped regions, Hypothesis 2b is accepted. The deeper reason may be that underdeveloped regions view environmental decentralization as an umbrella for economic development and tend to invest in non-green innovation areas that contribute more to GDP growth, thus curbing the development of green innovation in the region.

### 5.3. The Moderating Effect of Environmental Decentralization

In order to inspect whether environmental decentralization affects the regional green innovation promotion effect of environmental regulation, this study adopts Stata 16.0 to centralize the environmental regulation and environmental decentralization variables, and then substitutes them into model (4) for regression estimation; the results are shown in Table 4. The estimated coefficient of the interaction term between environmental regulation and environmental administration and supervision decentralization are significantly negative, indicating Hypothesis 3a is correct, which is similar to the findings of Lipscomb, M. et al. [79]. The interaction term between environmental monitoring decentralization and environmental regulation is significantly positive, suggesting Hypothesis 3b is not rejected. Under the increasing economic level, the technology and funding for environmental monitoring under environmental monitoring decentralization have been effectively secured, the authenticity of monitoring data has been greatly improved, and the focus of environmental regulation has been adjusted in a timely manner, thus improving the performance of regulation.

### 5.4. Further Analysis: Threshold Effect Test

#### 5.4.1. Number of Thresholds

Before using the panel threshold model, the sample needs to be tested for the presence of threshold effects to determine the number of thresholds. The model is estimated, the F-test statistic is obtained, and the probability values *p* and threshold values are calculated using a bootstrap method with 300 replicate samples as shown in Table 5. It can be seen that the results of the double threshold test pass the significance test with environmental decentralization as the threshold variable, and the two thresholds are 0.5941 and 0.9694 respectively.

#### 5.4.2. Threshold Regression Analysis

**Figure 2:**
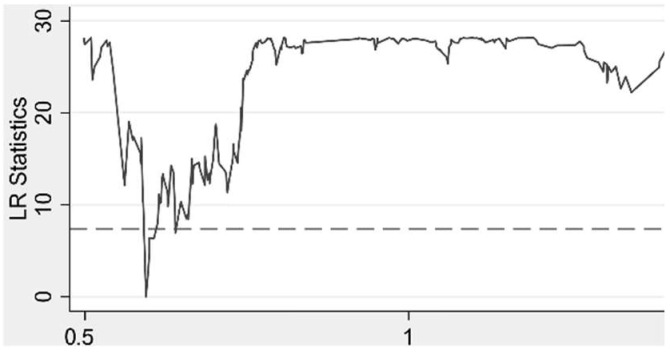
First Threshold.

**Figure 3:**
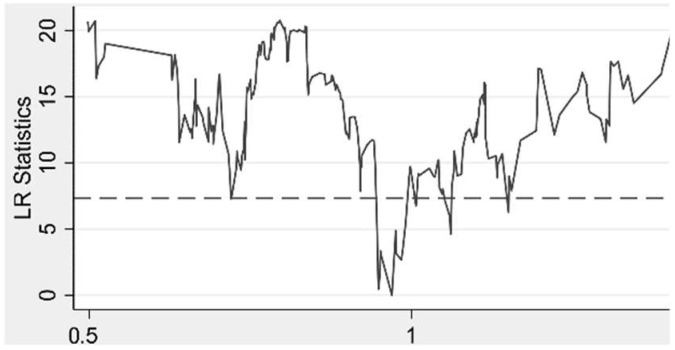
Second Threshold.

In order to determine whether the thresholds for environmental decentralization truly exist, the LR test was performed in this study and the results are shown in Figure 2 and Figure 3. It can be seen that both thresholds pass the LR test, i.e., both thresholds truly exist.

After the threshold values were determined, the parameters of the double threshold model were estimated according to Equation (5) and the estimation results are shown in Table 6. It can be seen that the impact of environmental regulation on green innovation indicates a nonlinear feature due to the environmental decentralization. Specifically, When ED ≤ 0.5941, the coefficient of environmental regulation on regional green innovation is 1.340, indicating that environmental regulation has a significant contribution to regional green innovation in this interval. When 0.5941 < ED ≤ 0.9694, the coefficient of environmental regulation on regional green innovation is 1.023, indicating that the environmental decentralization in this interval weakens the promotion of environmental regulation on regional green innovation to some extent. When 0.9694 < ED, the coefficient of environmental regulation on regional green innovation is 0.606, showing that excessive environmental decentralization significantly inhibits the regional green innovation effect of environmental regulation, which is similar to the findings of Yang et al. [80].

One possible reason for this finding is that when the environmental decentralization level is suitable, the decentralization of environmental power could help local governments to exert their information advantages and coordinate the allocation of funds and personnel in the process of environmental governance, thereby enhancing the green innovation of local enterprises [81,82]. When the environmental decentralization level is higher than the second threshold (0.9694), local governments have greater randomness in self-evaluation of regional environmental conditions [83,84]. To some extent, it provides an opportunity for collusion between local government and enterprises, which may reduce the enterprises’ incentives of technological innovation, energy saving, and emission reduction [85].

## 6. Conclusions and Policy Recommendations

Building a scientific and rational modern environmental management system is an institutional guarantee to achieve green development. How environmental management affairs are appropriately divided between the central and local governments closely affects the efficiency and quality of regional green innovation. Therefore, this paper analyzed environmental regulation, environmental decentralization, and regional green innovation under the same framework by constructing a fixed-effects model and a panel threshold model. The research draws the following conclusions. First, environmental regulation significantly promotes regional green innovation while environmental decentralization exacerbated regional innovation, and their effect coefficients are 1.112 and −0.761 respectively. Second, the impact of environmental regulation and environmental decentralization on green innovation is heterogeneous, with the level of economic development and the type of environmental decentralization being key factors influencing the role of environmental regulation. The result confirms that green innovation utility of environmental regulation increases with the improvement of regional economic level [86]. Finally, excessive environmental decentralization inhibits green innovation by hindering the effective implementation of environmental regulatory policies, and the biggest threshold of environmental decentralization is 0.9694. According to the conclusions, some policy implications can be obtained as follows.

First, the central government can appropriately increase the intensity of environmental regulations to increase the motivation of local governments to engage in green innovation. Currently, Chinese enterprises have the strength to withstand high standards of environmental regulations. The government should not only increase emission charges for heavy polluters, but also actively encourage enterprises to engage in green innovation activities such as clean technology research and development.

Second, under China’s environmental decentralization system, the local government’s attitude of sacrificing the environment for the economy is rooted in the imperfection of the performance appraisal system. Therefore, it is necessary to accelerate improving the good performance evaluation system, combining economic and environmental indicators. The setting of performance evaluation indicators should coordinate the relationship between economic growth and environmental governance. The government cannot sacrifice the environment for rapid growth, nor can it excessively pursue environmental quality at an economical cost [87].

Third, differentiated environmental decentralization strategies need to be developed for different regions. Since developed regions have stronger economic and technological strength, local environmental autonomy can be moderately relaxed to make full use of the information advantages of local governments so as to improve regional green innovation performance. For underdeveloped regions, the central government should reduce the environmental administrative and monitoring powers of local governments, while increasing environmental assessment and supervision, as well as setting environmental bottom-line standards and incentives to motivate local governments to conduct green innovation.

Although this study for the first time quantitatively investigates the relationship between environmental regulation, environmental decentralization, and regional green innovation, there are still some limitations, which could also be possible future research directions. For instance, the provincial panel data utilized in this study is not large due to the data availability, which may be prone to some small sample size bias. Moreover, the effect of environmental decentralization is closely related to the game and competition between local governments. In particular, environmental governance in neighboring areas may affect one another, thus the spatial spillover of environmental effects of environmental decentralization caused by local competition can be considered in future research.

## Figures and Tables

**Table 1:** Descriptive statistics for key variables.

Variables	Definition	Mean	Std. Dev.	Min	Max	Observations
**GI**	Green patent applications	2785	4180	14	28,049	300
**ER**	Environmental regulation	0.92	0.42	0.24	2.51	300
**ED**	Environmental decentralization	0.97	0.35	0.48	2.29	300
**EAD**	Environmental administrative decentralization	1.37	1.22	0.26	6.15	300
**ESD**	Environmental supervision decentralization	1.35	1.46	0.10	8.10	300
**EMD**	Environmental monitoring decentralization	1.53	1.49	0.22	7.75	300
**RGDP**	Gross National Product per capita	22,099	13,084	5787	66,036	300
**UR**	Urbanization rate	52.50	13.94	27.46	89.6	300
**TFI**	Total foreign investment	5224	7385	139	33,127	300
**IND**	Advanced industrial structure	41.57	8.68	28.3	79.7	300

**Table 2:** National level regression results.

Variables	ED	EAD	ESD	EMD
ER	1.112 ***	1.060 ***	1.027 ***	1.123 ***
	(0.142)	(0.142)	(0.145)	(0.148)
ED	−0.761 ***	−0.147 ***	−0.0983 ***	0.00758
	(0.164)	(0.0309)	(0.0231)	(0.0326)
lnRGDP	1.298 ***	0.920 ***	1.021 ***	1.208 ***
	(0.316)	(0.320)	(0.320)	(0.329)
lnUR	4.864 ***	5.226 ***	5.101 ***	4.824 ***
	(0.379)	(0.387)	(0.386)	(0.401)
lnIND	1.536 ***	1.385 ***	1.443 ***	1.604 ***
	(0.249)	(0.252)	(0.253)	(0.262)
lnTFI	−0.167 ***	−0.304 ***	−0.194 ***	−0.230 ***
	(0.0621)	(0.0632)	(0.0617)	(0.0719)
Constant	−29.66 ***	−26.27 ***	−27.84 ***	−29.16 ***
	(2.743)	(2.801)	(2.775)	(2.850)
Observations	300	300	300	300
R-squared	0.915	0.915	0.914	0.908

Note: *** indicates significance at the 1% level.

**Table 3:** Regional level regression results.

Variables	Developed Regions				Underdeveloped Regions			
	ED	EAD	ESD	EMD	ED	EAD	ESD	EMD
ER	0.902 ***	0.902 ***	0.978 ***	1.042 ***	0.484 *	0.489 *	0.527 *	0.574 **
	(0.207)	(0.199)	(0.209)	(0.221)	(0.267)	(0.269)	(0.272)	(0.275)
ED	−0.646 ***	−0.275 ***	−0.109 ***	−0.124 *	−0.847 ***	−0.0865 **	−0.0478	0.0558
	(0.191)	(0.0629)	(0.0355)	(0.0634)	(0.289)	(0.0364)	(0.0322)	(0.0372)
Control	YES	YES	YES	YES	YES	YES	YES	YES
Constant	−40.58 ***	−33.04 ***	−29.94 ***	−33.43 ***	−25.41 ***	−23.94 ***	−25.67 ***	−25.11 ***
	(5.920)	(5.757)	(6.475)	(6.539)	(3.034)	(3.130)	(3.089)	(3.100)
Observations	100	100	100	100	200	200	200	200
R-squared	0.937	0.942	0.936	0.932	0.915	0.914	0.912	0.912
Number	10	10	10	10	20	20	20	20

Note: ***, **, and * indicate significance at the 1%, 5%, and 10% levels, respectively.

**Table 4:** Regression results for moderating effects.

Variables	ED	EAD	ESD	EMD
c_ER	0.942 ***	1.030 ***	0.818 ***	1.045 ***
	(0.154)	(0.143)	(0.155)	(0.151)
c_ED	−0.807 ***	−0.160 ***	−0.163 ***	0.00134
	(0.164)	(0.0315)	(0.0298)	(0.0325)
c_EDER	−0.858 ***	−0.132*	−0.245 ***	0.183 **
	(0.323)	(0.0705)	(0.0727)	(0.0779)
Control	YES	YES	YES	YES
Constant	−29.94 ***	−25.85 ***	−26.27 ***	−27.86 ***
	(2.800)	(2.851)	(2.794)	(2.900)
Observations	300	300	300	300
R-squared	0.917	0.916	0.917	0.910

Note: *** and ** indicate significance at the 1% and 5% levels, respectively.

**Table 5:** Threshold quantity test and threshold estimation.

Variables	Threshold Number	F-Value	*p*-Value	Threshold	95% Confidence Interval
ED	Single threshold	30.98	0.0100	0.5941	(1.0656, 1.6146)
	Double Threshold	21.26	0.0767	0.9694	(0.7426, 1.3043)
EAD	Single threshold	22.93	0.0967	3.8584	(0.8714, 1.4276)
	Double Threshold	7.17	0.7433	2.3283	(0.6477, 1.2827)
ESD	Single threshold	14.64	0.3800	3.0288	(0.6340, 1.2633)
	Double Threshold	19.68	0.1233	0.6851	(0.4037, 1.0421)
EMD	Single threshold	12.43	0.4033	2.3724	(0.7500, 1.3437)
	Double Threshold	8.60	0.6367	1.0269	(0.5801, 1.1841)

**Table 6:** Coefficient estimation results of the threshold model.

Variables	lnRGDP	lnUR	lnIND	lnTFI	$\alpha_{1}$	$\alpha_{2}$	$\alpha_{3}$
(5)	1.471 ***	−0.174 ***	1.629 ***	4.804 ***	1.340 ***	1.023 ***	0.606 ***
	(0.306)	(0.0587)	(0.238)	(0.363)	(0.139)	(0.143)	(0.158)

Note: *** indicates significance at the 1% level.

## Data Availability

The data presented in this study are available on request from the corresponding author. The data are not publicly available due to data management.
